# Repurposing Drugs in Oncology (ReDO)—Propranolol as an anti-cancer agent

**DOI:** 10.3332/ecancer.2016.680

**Published:** 2016-10-12

**Authors:** Pan Pantziarka, Gauthier Bouche, Vidula Sukhatme, Lydie Meheus, Ilse Rooman, Vikas P Sukhatme

**Affiliations:** 1 Anticancer Fund, Brussels, 1853 Strombeek-Bever, Belgium; 2 The George Pantziarka TP53 Trust, London, UK; 3 GlobalCures, Inc, Newton MA 02459, USA; 4 Oncology Research Centre, Vrije Universiteit Brussel, 1090 Brussels, Belgium; 5 Beth Israel Deaconess Medical Center and Harvard Medical School, Boston, MA 02215, USA

**Keywords:** Propranolol, beta-blockers, drug repurposing, ReDO project

## Abstract

Propranolol (PRO) is a well-known and widely used non-selective beta-adrenergic receptor antagonist (beta-blocker), with a range of actions which are of interest in an oncological context. PRO displays effects on cellular proliferation and invasion, on the immune system, on the angiogenic cascade, and on tumour cell sensitivity to existing treatments. Both pre-clinical and clinical evidence of these effects, in multiple cancer types, is assessed and summarised and relevant mechanisms of action outlined. In particular there is evidence that PRO is effective at multiple points in the metastatic cascade, particularly in the context of the post-surgical wound response. Based on this evidence the case is made for further clinical investigation of the anticancer effects of PRO, particularly in combination with other agents. A number of trials are on-going, in different treatment settings for various cancers.

## Introduction

Propranolol (PRO) is a commonly used non-selective beta-adrenergic receptor antagonist used in the treatment of hypertension, angina, anxiety, cardiac arrhythmia, hyperthyroidism, essential tremor and as a prophylaxis against migraine, variceal bleeding and myocardial infarction. First developed in the 1960s, the drug is now available globally in generic form and is on the WHO List of Essential Medicines. The drug is available in both standard and extended-release tablet formulations, as an oral solution and also as intravenous injection. Common trade names include Inderal, Angilol, Syprol, Ciplar. A special oral formulation, (Hemangiol in Europe, Hemangeol in the USA), has also been licensed by the EMA and FDA, for the treatment of infantile hemangioma. There is also clinical trial data supporting the off-label use of PRO in a number of conditions, including haemorrhage, sepsis and hypermetabolic syndrome associated with severe burns [1], akathisia associated with Alzheimer’s disease or psychosis [2], and aggression associated with brain injury or disease [3].

In addition to clinical use in these varied indications there is accumulating evidence that propranolol has potent anti-cancer effects, as evidenced by in vitro, in vivo and a range of clinical data.

## Current Usage

### Dosage

The PRO dose varies by indication. The anti-hypertensive dose is in the range 160 – 320 mg/day, starting at 80 mg and increasing as required to a maintenance dose that is generally 160 mg – 240 mg, in divided doses or as once a day use of extended release tablets. For angina the dose is 120 – 240 mg/day. Migraine prophylaxis is in the range 80 – 240 mg/day [4]. The dose in infantile hemangioma is 1 mg/kg/day for 1 week, then 2 mg/kg/day for 1 week and then 3 mg/kg/day as a maintenance dose for 6 months.

### Toxicity

Common side effects include insomnia, fatigue, cold extremities and Raynaud’s syndrome. Less common side effects include nausea, vomiting, diarrhoea. Rarely PRO is associated with heart failure, heart block, hypotension, worsening of symptoms in psoriasis, asthma and psychosis. In general the initiation of PRO may lead to initial, mild adverse effects which resolve during dose titration to a maintenance dose. Sudden termination of treatment is not advised, particularly for patients suffering ischaemic heart disease – in such cases the dose should be tapered rather than stopped abruptly. In general however, PRO has a good toxicology profile and can be used for long-term treatments of many years duration [5].

PRO is contra-indicated in patients suffering from hypotension, asthma, uncontrolled heart failure, severe peripheral artery disease, metabolic acidosis and cardiogenic shock. It is not recommended in pregnancy and during lactation.

### Pharmacokinetics

PRO is highly lipophilic and undergoes rapid absorption in the gastrointestinal tract and more than 90% undergoes plasma protein absorption. Excretion is primarily renal, though 1 – 4% of an oral or IV dose of the drug appears in faeces as unchanged drug and metabolites [6]. There is wide distribution to tissues, particularly lungs, liver, kidneys, and heart. Bioavailability after oral dosing is in the range 25 – 35% due to extensive first-pass hepatic clearance, although there is considerable inter-patient variability [7]. Bioavailability can be increased by concomitant food intake, with a mean increase of around 50% reported when taken after a protein-containing meal, however other parameters (time to maximum concentration, half-life etc.) are unchanged [8].

Peak plasma concentrations occur 1.5 – 3 hours following oral dosing, with a plasma half-life of around 4 hours following single dose or around 10 hours for extended release tablets. Mean peak plasma concentration following a single oral dose of 40 mg, in fasting conditions, is 38 ng/ml (0.12 μM) [9]. A single oral dose of 160 mg produced a peak in the range 200 – 245 ng/ml (0.77 – 0.96 μM), while the extended release tablets produced a peak in the range 18 – 50 ng/ml (0.07 – 0.19 μM) [10]. PRO crosses the blood brain barrier [11]. There is some evidence that the effects of PRO are dependent on the plasma concentrations that are achieved, including in the treatment of rare benign tumours in children [12].

Hepatic metabolism of PRO involves multiple pathways in the cytochrome P450 system (CYP2D6, 1A2 and 2C19), and therefore a range of drug interactions are possible [4, 6]. For example, concurrent cimetidine increased the area under the curve (AUC) and doubled peak plasma levels of PRO [13].

Caution is advised when PRO is used with calcium-channel blocking drugs, particularly IV verapamil, in patients with severe cardiomyopathy, congestive heart failure, or recent myocardial infarction due to the negative inotropic and chronotropic action of these drugs [14].

## Pre-clinical Evidence in Cancer - *In Vitro* and *In Vivo*

Investigation of the possible anticancer properties of PRO began in the late 1970s, primarily with regards to elucidating the roles of catecholamines in carcinogenesis and in identifying beta-adrenergic receptor binding sites in different tissues [15–17].

### Leukaemia

One of the first findings that PRO may be of some benefit in cancer treatment was reported by Ramu *et al*, who, in 1984, published a report that looked at the activity of a range of drugs in reversing in vitro drug resistance in the P388/ADR murine leukaemia cell line [18]. PRO was shown to have moderate effects in restoring sensitivity to doxorubicin in P388/ADR cells, but showed no evidence of additional effects in the parental P388 cell line. Similarly, Tsuruo *et al* showed that 10 μM of PRO significantly (P < 0.05) enhanced the cytotoxicity of vincristine and doxorubicin in resistant P388/VCR and P388/ADR cell lines respectively [19]. Other investigators have also reported similar effects in reversing resistance in multi-drug resistant human epidermoid KB carcinoma cell lines [20].

Hajighasemia and Mirshafiey investigated the cytotoxicity of PRO against Molt-4 and Jurkat human leukaemia and the U937 monocyte cell lines [21]. They showed that the viability of all three cell lines was dose- and time-dependently reduced by PRO above concentrations of 200 μM.

An investigation by Lamkin *et al* in a murine model of acute lymphoblastic leukaemia (ALL) found that chronic stress enhanced tumour growth and dissemination and that the effect could be inhibited by PRO [22]. PRO has also been shown to inhibit the expression of the tissue remodelling factor matrix metalloproteinase-2 (MMP-2) and the pro-angiogenic vascular endothelial growth factor (VEGF) in human leukaemia cell lines [23].

### Breast

Shakhar and Ben-Eliyahu reported on the influence of beta adrenergic agonists and antagonists on natural killer (NK) cell number and activity in F344 rats inoculated with highly metastatic MADB106 syngeneic mammary adenocarcinoma cells [24]. Injection of the beta-adrenergic agonist metaproterenol (MP) led to a transient increase in NK cell numbers which returned to baseline within one hour; however, there was a concomitant decrease in NK activity over the same period. The beta-adrenergic antagonists nadolol and PRO were able to suppress these effects. Furthermore, treatment with MP was associated with a 10-fold increase in the number of injected tumour cells retained in the lungs 1-day post-inoculation, and a corresponding increase in pulmonary metastatic lesions three-weeks post-inoculation. These effects were dose-dependent and reversible by co-treatment with the non-selective beta-blocker nadolol.

Benish *et al* investigated the effect of inhibiting cyclo-oxygenase-2 (COX-2) and of blocking beta-adrenergic receptors on post-surgical immune function and metastatic tumour growth [25]. F344 rats underwent laparotomy and were injected with syngeneic MADB106 cells. Rats pre-treated with COX-inhibitors (SC560, indomethacin, etodolac, or celecoxib) or vehicle were compared to non-surgically treated controls to assess the impact on tumour cell retention in the lungs (LTR), and it was shown that all surgically treated animals had an elevated rate of LTR compared to non-surgically treated, but that COX-2 inhibition (indomethacin, etodolac, or celecoxib, administered one hour prior to surgical incision) significantly attenuated the increase compared to the vehicle treated group. Other experiments combined etodolac and PRO, (both administered one hour prior to incision), and again showed that LTR was reduced by either treatment alone and in combination, and that chronic and acute treatment had similar outcomes. Finally, it was shown that surgery was also associated with reduced NK cell cytotoxicity, which could also be reversed by the combination of etodolac and PRO. Subsequent work by the same group reproduced similar results and additionally showed that combination treatment with the immunostimulant CpG-C increased the effect of the PRO + etodolac combination [26].

The effect of sympathetic nervous system signalling was investigating in a murine breast cancer model by Sloan *et al* [27]. BALB/c mice were subjected to two hours per day of restraint, shown previously to induce catecholamine-mediated stress, or control conditions for five days prior to injection of syngeneic 66cl4 mammary carcinoma cells. Stressed animals showed reduced weight and a 38-fold increase in the rate of metastasis (P = 0.04), both in terms of increased number and size of metastases compared to unstressed controls. Increased stress was not associated with significant changes in primary tumour growth. PRO treatment had no effect on metastatic growth in control animals but completely inhibited the enhanced increase in metastases in stressed animals (P < 0.001). Others have also investigated the role of norepinephrine in the metastatic process and reported a similar anti-metastatic activity of PRO in murine models of breast cancer [28–29].

Having first ascertained that low concentrations of PRO and 5-FU or paclitaxel increased the anti-proliferative and anti-angiogenic effects of these standard chemotherapeutic drugs in a panel of cancer and non-cancer cell lines. Pasquier *et al* studied the in vivo effects of the combination in NMRI nude mice orthotopically injected with MDA-MB-231 human triple negative breast cancer cells [30]. PRO, at a dose of 10 mg/kg, with paclitaxel increased median survival by 79% compared to paclitaxel alone (P = 0.0005) and PRO with 5-FU increased median survival by 19% (P = 0.0005).

The role of beta-adrenergic signalling in breast cancer metastasis to the bone was investigated by Campbell *et al* [31]. In vitro experiments showed that beta-adrenergic signalling upregulated RANKL expression in osteoblasts and that this increased MDA-MB-231VU human mammary carcinoma cell migration. An in vivo model, using the same cell line in athymic mice, showed that chronic stress or exogenous beta-adrenergic agonist isoproterenol increased both the number and area of osteolytic lesions compared to controls and that PRO, supplied *ad libitum* (0.5 g/L) via drinking water, could reverse this increase (P < 0.05).

In HER2-amplified breast cancer, Liu *et al* investigated the role of catecholamines and PRO on resistance to trastuzumab [32]. After showing a strong association between trastuzumab-resistance and beta2 adrenergic signalling in patient tissue samples, they showed that the catecholamines epinephrine and isoproterenol antagonised the anti-proliferative effect of trastuzumab both in vitro and in vivo. Additionally they showed that PRO could inhibit this antagonist effect, and resensitise resistant cells, both in vitro and in a xenograft model (PRO dosed at 2 mg/kg).

### Melanoma

The impact of psychosocial stress on cancer growth in two murine model of cancers was studied by Hasegawa and Saiki [33]. Groups of mice were housed in different conditions to simulate the effects of social crowding on growth of syngeneic tumours (B16 melanoma in C57BL/6 mice and Meth A fibrosarcoma in BALB/c mice). Three housing conditions were used – isolated, grouped and over-crowded – and the effect on tumour growth assessed. B16 melanoma growth rates were increased most in the order over-crowded, isolated and grouped animals. Additionally another over-crowded cohort was administered PRO, at a dose of 30 ppm from days -21 to +21 after tumour implantation. This cohort showed reduced tumour growth dynamics, with the initial growth rate lower than the grouped (non-stressed) cohort and was significantly lower than either of the stressed groups. Organ weight was also assessed and a negative correlation detected between thymic mass and tumour mass (P < 0.05). Repeated experiments compared the effects of over-crowding versus isolation in additional cohorts of melanoma-bearing C57BL/6 mice and in sarcoma-bearing BALB/c mice. Over-crowding was shown to be more strongly associated with increased tumour growth and thymic atrophy than isolation.

Dal Monte *et al* investigated the role of beta3 adrenergic receptors in melanoma growth and vascularisation [34]. In some of the in vitro experiments PRO was used alongside two selective beta3 adrenergic receptor antagonists, SR59230A and L-748,337. PRO, at a concentration of 10 μM, with and without exogenous norepinephrine, significantly reduced proliferation (P < 0.001) and increased apoptosis (P < 0.01) of B16F10 cells compared to controls. Wrobel and Le Gal also showed that PRO had significant effects on proliferation and apoptosis in vitro on a panel of melanoma cell lines at a high concentration of 100 μM [35]. In vivo experiments using both primary and metastatic human melanoma tumours transplanted into immunodeficient (Nod SCID Gamma) mice showed that PRO at an average dose of 1.7 mg/day, tumour volumes were significantly lower (P < 0.001) than in untreated controls.

Glasner *et al* investigated the effects of surgical intervention on survival in two syngeneic mouse models, and on the impact of pre-operative PRO and etodolac, a COX-inhibitor, on survival [36]. B16 melanoma-bearing C57BL/6J mice were administered PRO, etodolac, PRO + etodolac, or vehicle 30-minutes prior to amputation and/or laparotomy. Treatment with either drug singly showed no statistically significant difference with vehicle in terms of survival for any of the surgical options, whereas combined PRO + etodolac significantly improved survival rates (P = 0.0345). In a Lewis Lung carcinoma model mice were pre-treated with IL-12 or vehicle 24-hours prior to amputation, treatment groups were further subdivided and treated with PRO + etodolac or vehicle prior to surgery. All treatment groups showed significant increases in survival rates, although there were no differences in effect sizes between IL-12, IL-12 + PRO + etodolac and PRO + etodolac treatments.

### Ovarian

Lutgendorf *et al* noted that ovarian cancer patients with greater levels of social isolation and distress had greater levels of serum VEGF, associated with increased angiogenesis, in contrast to patients with lower levels of social isolation [37]. Subsequent in vitro investigation using the SKOV3 and EG ovarian carcinoma cell lines showed that norepinephrine, epinephrine, isoproterenol (a nonspecific beta-adrenergic agonist), and cortisol enhanced the production of VEGF in both cell lines. Pre-treatment with PRO, at a concentration of 1 μM, abolished this increase in VEGF [38]. Later work showed that in a murine SKOV3 model PRO was able to reduce isoproterenol-induced tumour growth [39].

Subsequent investigation showed that surgical stress, from a wound distant from the implanted tumour, was associated with increased primary tumour growth rate and the multiplicity of metastases in a two murine ovarian cancer models (HeyA8 and SKOV3ip1) but not in beta-adrenergic receptor-negative RMG-II mice [40]. Treatment with PRO via micro-osmotic pump starting seven days prior to surgical intervention inhibited the post-surgical increase in tumour growth rate and reduced the number of metastatic nodules.

### Angiosarcoma

In light of the positive clinical experience of PRO in the treatment of infantile hemangioma, most recently confirmed in a large multi-centre randomised controlled trial [41], and evidence of beta-adrenergic receptor expression in a range of vascular tumours [42], a number of investigators have explored the potential benefit of PRO. Stiles *et al* used in vitro and in vivo models of hemangioendothelioma and angiosarcoma to investigate the impact of beta-blockade with PRO on cell proliferation, migration and apoptosis [43]. Using a panel of canine angiosarcoma, murine angiosarcoma and murine hemangioendothelioma cell lines, it was shown that 25 μM of PRO inhibited proliferation in all lines compared to untreated controls (P < 0.05). At a higher concentration of 100 μM, PRO induced apoptosis in all cell lines (P < 0.05), and showed synergistic action in cells treated with chemotherapy (cisplatin, busulfan, vincristine, or H2O2) with the exception of the murine hemangioendothelioma cell line. Finally, in a murine model of angiosarcoma PRO treatment at a dose of 20 mg/kg every 2 days led to a significant reduction of tumour growth compared to controls (tumour weight of 357+/-58 mg; N = 17 vs sham 984+/-92 mg; N = 15, P < 0.0001). Despite the reduction in tumour size, tumour sections from both sham and PRO conditions revealed active cell division, suggesting the need to employ combinatorial therapy with PRO.

Pasquier at al showed that immortalised and NRAS-transformed endothelial cells were sensitive to the anti-proliferative effects of PRO [44]. Furthermore, PRO was shown to have antagonistic or additive effects when combined with doxorubicin or paclitaxel, commonly used to treat angiosarcoma, but that the effect was synergistic in combination with vinblastine. In a 3D *in vitro* model BMST-Ras cells were allowed to form spheroids of ~600 μm in diameter before treatment was initiated. When used alone, 10 μM propranolol and 1 nM vinblastine significantly slowed down the growth of tumour spheroids, resulting in a 19 – 20% decrease in volume after 5 days of treatment as compared to untreated spheroids (P < 0.001). The combination of propranolol and vinblastine completely suppressed the growth of tumour spheroids, leading to a 59% decrease in volume after 5 days as compared to control spheroids (P < 0.001).

### Neuroblastoma

Pasquier *et al* tested a panel of seven beta-adrenergic antagonists, alone and in combination with vincristine, on BE(2)-C and SHEP neuroblastoma cell lines in vitro [45]. Of the seven drugs tested the most potent anti-proliferative and anti-angiogenic agents were carvedilol, nebivolol and PRO. While these three agents did not significantly impact on the anti-cancer effects of a range of chemotherapeutics, they showed significant (P < 0.001) synergy with the vinca alkaloid vincristine. Similar results were obtained with vinblastine and paclitaxel, suggesting the effect was related to microtubule disruption. In vivo results, using a TH-MYCN transgenic mouse model of neuroblastoma, showed that PRO, at a dose of 50 mg/kg i.p., increased the rate of tumour regression induced by vincristine treatment (P < 0.05). Finally, analysis of survival times showed that mice treated with PRO and vincristine had a fourfold increase in median survival compared to treatment with vincristine alone (P < 0.0001). Carvedilol with vincristine seemed the most effective combination and resulted in sustained complete remission in one animal (of ten), which remained tumour-free until study completion (day 60).

A xenograft model of paediatric neuroblastoma, based on the BE(2)-C cell line, was used by Xu *et al* [46]. Animals with established tumours were treated with different doses of PRO – 2, 5 or 10 mg/kg – for nine days and compared to untreated controls. Animals were sacrificed on day 9 and tumour weights in the 2 mg/kg and 5 mg/kg groups were 36.6% (P < 0.002) and 34.4% (P < 0.005) lower than the control group. Tumour weights in the 10 mg/kg were 18.3% lower than control, a figure not statistically significant. Additional analyses showed that microvessel density (MVD) was lower in the treated groups than in controls (P < 0.01) and VEGF, MMP-2, and MMP-9 protein levels were significantly lower 5 mg/kg and 10 mg/kg groups (P < 0.05).

Wolter *et al* tested PRO against a panel of 15 neuroblastoma cell lines representing a range of genetic profiles [47]. PRO inhibited cell growth, reducing proliferation and viability, in all lines at IC50 values in the range 114 μM to 218 μM. It was also shown to be synergistic with SN-38, the active metabolite of irinotecan, with significantly reduced viability compared to treatment with either agent alone (P = 0.008 for PRO alone, P = 0.0009 for SN-38 alone). Using SK-N-AS cells in xenograft models, treatment with PRO at 2 mg/kg/day injected subcutaneously produced lower tumour volume at day 14 compared to controls, (P = 0.0246) and was associated with longer median survival (P = 0.0135).

### Prostate

Work by Masur *et al* showed that norepinephrine, at a concentration of 10 μM, increased the migratory activity of PC3 human prostate carcinoma cells in vitro but had no influence on proliferation levels, and that PRO at the same dose significantly inhibited this increase [48]. Furthermore, athymic BALB/c mice injected with PC3 cancer cells were treated with norepinephrine, PRO or a combination, administered via micro-osmotic pumps, and the rate of primary and metastatic tumour growth was assessed. While neither treatment had significant effect on primary tumour growth, norepinephrine was associated with a significant increase (P = 0.014) in metastatic tumour growth compared to controls, and PRO treatment reduced metastatic tumour growth below controls (P = 0.009).

Brohée *et al* showed that in vitro PRO concentrations of 100 μM potentiated the anti-proliferative effect of rapamycin on human prostate cancer PC3 cells [49].

### Pancreatic

Guo *et al* showed that PRO inhibited MMP-2, MMP-9 and VEGF in pancreatic cancer cell lines [50]. Zhang *et al* also reported that PRO was able to induce apoptosis in the PC-2 human pancreatic cancer cell line at concentrations of 100 μM [51], and reduce invasiveness in MIA PaCa-2 and BxPC-3 cell lines at the same concentration [52].

Kim-Fuchs *et al* used Panc-1 human pancreatic cancer cells to establish orthotopic tumours in BALB/c-Foxn1nu nude athymic mice [53]. Mice were subject to restraint or home cell conditions to mimic stress or control respectively. Stress, verified by changes in body weight and tissue catecholamine levels, was associated with an increased rate of pancreatic tumour growth by 11% ± 3 per day compared to control mice (P < 0.01) and increased tumour mass five-fold (7.5 mg ± 5 vs. 41 mg ± 13, P = 0.03). Additionally chronic stress was associated with metastatic spread in 50% of mice, compared to none in the controls. PRO treatment, via osmotic pump delivering 10 mg/kg/day, blocked the effect of stress on primary tumour growth (41 mg ± 13 vs. 21 mg ± 5), although there was no change in the metastatic rate in the time-frame of the experiment.

Partecke *et al* also investigated the impact of chronic stress on pancreatic cancer growth using C57BL/6 mice bearing orthotopic syngeneic 6606PDA tumours [54]. Stress was shown to be associated with immunosuppression and an increase in tumour angiogenesis and cancer cell invasion. Median survival in stressed mice was significantly reduced compared to unstressed control mice (52 days versus 66 days, P < 0.0058). Treatment with PRO, (given orally via drinking water using a concentration of 0.5 g/l of drinking water aiming at 30 – 60 mg/kg mouse/day), reduced tumour growth rates and increased survival compared to untreated controls (59 days versus 52 days in controls, P < 0.0058).

### Colorectal

Masur *et al* investigated the effect of norepinephrine and PRO on the in vitro migratory activity of SW 480 colon carcinoma cells [55]. Where norepinephrine caused a dose dependent increase in the number of migrating cells above basal levels, treatment with PRO at the same concentrations (1 μM, 10 μM and 100 μM) abolished this increase. In contrast treatment with the selective beta1 adrenoreceptor antagonist atenolol did not interfere with the norepinephrine-induced increase in migratory cell numbers. Similarly, Coelho *et al* explored the impact of a number of beta adrenergic receptor agonists and antagonists on the proliferation of HT-29 colon adenocarcinoma cells [56] and reported the IC50 of PRO as 65.4 μM.

Chin *et al* also investigated the effect of selective beta2-adrenergic receptor antagonists, including PRO, in a panel of colorectal cancer cells lines [57]. They showed that PRO significantly inhibited the viability of SW620, Colo205, and HT29 cells (IC50 119.5, 86.38, and 69.1 μM, respectively). PRO induced G1-phase arrest and apoptosis in affected cell lines.

Lin *et al* showed that chronic restraint stress promoted tumour growth in xenograft models of colorectal cancer, and that this effect could be blocked, in vivo, using PRO at a dose of 2 mg/kg [58].

### Head and Neck

Yang *et al* investigated the effects of norepinephrine on nasopharyngeal carcinoma cell lines (HONE-1, HNE-1, and CNE-1) [59]. Treatment of HONE-1 cells with norepinephrine upregulated levels of metalloproteinases (specifically MMP-2 and MMP-9) and VEGF and increased cell invasiveness. Treatment with PRO inhibited this increase in MMP-2, MMP-9 and VEGF. Inhibition by PRO of MMP-9 secretion has also been confirmed in human brain microvascular endothelial cells [60].

Bernabé and colleagues assessed the influence of norepinephrine and cortisol on oral squamous cell carcinoma cell lines (OSCC) [61]. They showed that norepinephrine and cortisol, at physiologically relevant levels, induced IL-6 production in SCC9, SCC15, and SCC25 cells and similar observations were made for isoproterenol in SCC9 and SCC25 cells. These effects were associated with an increase in cell proliferation and PRO, at a concentration of 1 μM, blocked this increase. Wolter *et al* also showed that PRO reduced the viability of SCC9, SCC17a, SCC25, and FaDu cell lines, and that it synergised with cisplatin and radiotherapy in treating SCC17a cells [62].

### Other

Grzanna and co-workers showed, in vivo, that administration of PRO delayed tumour growth of LPC-1 plasmacytoma tumours in female BALB/c mice [63]. Mice were treated with doses of 0.6. 6 and 60 mg/kg/day, administered via osmotic pumps, and compared with untreated mice following subcutaneous injection of LPC-1 cells. All treated mice showed delayed tumour growth compared to untreated controls, in a dose-dependent manner. There was also a dose-dependent decrease in IgG plasma values, confirming the effect seen on tumour size. However, while treatment increased tumour growth latency, there was no difference in growth rates once tumours were established.

The effect of PRO on tumour perfusion was investigated by Bomber *et al* in a small 1986 study focused on enhancing uptake of Ga-67 to improve imaging of small tumours [64]. PRO was administered, at a dose of 10 mg/kg, 10 minutes prior to administration of radiolabelled gallium citrate. Analysis showed that PRO doubled tumour perfusion and decreased Ga-67 uptake in non-tumour tissues, thereby increasing the relative uptake in the tumour compartment. Subsequently Burton and Gray reported that a combination of norepinephrine and PRO enhanced the relative blood supply to hepatic tumours in rabbits and doubled the embolisation rate of microspheres in the tumour compartment (P < 0.05) compared to either norepinephrine or PRO alone [65].

The effect of PRO was also investigated in two human gastric carcinoma cell lines (SGC-7901 and BGC-823) by Liao and colleagues [66]. In vitro concentrations of 200 μM induced cell cycle arrest and apoptosis in both cell lines. The effect of PRO on the radiosensitivity of the same gastric carcinoma cell lines was also investigated [67]. When pre-treated for 24 hours with PRO at a concentration of 50 μM both cell lines displayed a significant increase in radiosensitivity and apoptosis. PRO was associated with a decreased level of NF-κB and down-regulation of VEGF, COX-2, and EGFR expression.

In non-small cell lung cancer (NSCLC), Al-Wadei and colleagues investigated the effects of chronic exposure to nicotine on cancer cell proliferation [68]. Results showed that nicotine-treated lung adenocarcinoma cells, (NCI-H322, NCI-H441 and NCI-H1299), released norepinephrine and increased proliferation. Cells treated with PRO at a concentration of 1 μM for 10-minutes prior to acute or chronic exposure to nicotine showed significant reductions in the number of viable cells compared to nicotine alone (P < 0.0001). Interestingly, exposure to PRO also reduced viability compared to cells not treated with nicotine. The same authors subsequently extended this line of research and showed a similar nicotine-drive autocrine catecholamine feedback loop in a pancreatic adenocarcinoma model [69].

Kozanoglu *et al* investigated the in vitro effect of PRO on the U266 human multiple myeloma cell line [70]. They showed a dose and time dependent effect on cell proliferation and apoptosis, with IC50 values of 141, 100, and 75 μM after 24-, 48-, and 72-h PRO exposure, respectively.

Abdi and colleagues used two doses of PRO, 5 mg/kg and 10 mg/kg, in mice bearing solid Ehrlich carcinoma tumours [71]. While both doses were associated with reductions tumour growth rates and volumes compared to untreated controls, it was only the higher dose group that was associated with improved survival (P < 0.05). Both dosage levels were associated with statistically significant reductions in IL-10, HSP70 and iNOS levels.

Wei *et al* explored the use of PRO in a panel of human thyroid cancer cell lines [72]. PRO was shown to inhibit growth of 8505C and K1 cell lines, with IC50 values of 200 μM and 280 μM, respectively. Growth inhibition was further analysed in 8505C line was shown to be associated with increased levels of apoptosis. In vivo data from a xenograft mouse model using 8505C cells and treated with PRO at a dose of 10 mg/kg showed reduced tumour volume increase compared to controls, with reduced SUVmax on PET/CT and Ki76 staining of tumour cells.

von Hippel-Lindau (VHL) disease, or von Hippel-Lindau syndrome, is a rare genetic disorder caused by a germ-line mutation in the von Hippel–Lindau tumour suppressor gene. It is characterised by the development of a range of tumour types, including hemangioblastoma of the retina and the central nervous system. Albiñana *et al* explored the effect of PRO on hemangioblastoma cells from VHL patients and reported a dose-dependent decrease in viability and increase in apoptosis [73]. This effect was enhanced in hypoxic conditions and was associated with a decreased the expression of HIF target genes, including VEGF.

## Human Data

The earliest human data to suggest a positive effect of propranolol on cancer came from epidemiological studies comparing cancer incidence in hypertensive and non-hypertensive patients. For example Perron *et al* reported on the incidence of prostate cancer in Canadian men treated with a range of anti-hypertensive drugs and found that treatment with beta-blockers had a protective effect with an adjusted odds ratio (OR) of 0.86 (95% CI = 0.77– 0.96) [74]. In patients with hepatitis C associated cirrhosis Nkontchou *et al* reported that PRO use was associated with a decreased risk hepatocellular carcinoma (HCC), with a hazard ratio (HR) = 0.25; (95% CI = 0.09 – 0.65; P < 0.004) [75]. Chang *et al* published a large cohort study of over 24 238 patients, with a PRO group (> 6 months use, n = 12 119) compared to a non-PRO use group (n = 12 119) over a 12 year period [76]. Overall the risk of cancer was lower in the PRO group (HR = 0.75; 95% CI = 0.67–0.85; P < 0.001), and site specific analysis showed a decreased risk in head and neck cancers (HR = 0.58; 95% CI = 0.35–0.95), oesophagus (HR = 0.35; 95% CI = 0.13 – 0.96), stomach (HR = 0.54; 95% CI = 0.30 – 0.98), colon (HR = 0.68; 95% CI = 0.49 – 0.93), and prostate cancers (HR = 0.52; 95% CI = 0.33 – 0.83).

Zhong *et al* performed a systematic review and meta-analysis of observational studies of beta-blocker use and cancer mortality [77]. They identified 24 relevant studies that had reported results by May 2015, involving 76 538 patients. Post-diagnosis use of a beta-blocker was associated with a statistically signification reduction in the risk of all-cause mortality (HR = 0.89; 95% CI = 0.81 – 0.98; P = 0.02). When stratified by cancer type the reduction in risk of death was only significant for breast cancer (HR = 0.82; 95% CI = 0.68 – 0.99; P = 0.03). In terms of cancer-specific mortality, beta-blocker use was associated with a reduced risk of cancer-specific mortality (HR = 0.89; 95% CI = 0.79 – 0.99; P = 0.03). However on stratification by cancer type there was no beneficial effect of post-diagnosis use of beta-blockers for breast, colorectal or prostate cancer. For pre-diagnosis use of beta-blockers there was no significant effect on all-cause mortality, but stratification by cancer type showed both a benefit for melanoma (HR = 0.81; 95% CI = 0.67 – 0.97; P = 0.02) and an increased risk for ovarian cancer (HR = 1.17; 95% CI = 1.04 – 1.32; P = 0.01). However, Weberpals *et al* evaluated a possible immortal time bias in these observational studies and found no clinically meaningful evidence for an association between beta-blocker use and survival when restricting the analysis to studies not prone to immortal time bias. Careful interpretation of observational studies is therefore required when no attempt is made to address immortal time bias [78].

### Breast Cancer

Results published by Barron *et al* in a larger population of breast cancer patients showed protective effects associated with PRO use [79]. Irish women treated with PRO (n = 70) or atenolol (n = 525) in the year prior to breast cancer diagnosis were matched with women not receiving beta-blocker treatment (n = 4738) in the ratio 1:2. PRO use was associated with a lower risk of presenting with a T4 (OR = 0.24, 95% CI = 0.07 – 0.85) or N2/N3/M1 (OR = 0.20; 95%C = 0.04 – 0.88) diagnosis compared to matched non-users. The cumulative probability of breast cancer-specific mortality was also significantly lower for PRO users compared to matched nonusers (HR = 0.19; 95% CI = 0.06 – 0.60). There was no difference in either outcome between atenolol users and matched non-users.

A systematic review and meta-analysis of beta-blocker use and breast cancer by Childers *et al* found a non-significant reduction in breast cancer recurrence (HR = 0.67; 95% CI = 0.39 - 1.13), a significant reduction in the risk of breast cancer mortality (HR = 0.50; 95% CI = 0.32 - 0.80) and no impact of all-cause mortality (HR = 1.02; 95% CI = 0.75 - 1.37) [80].

### Angiosarcoma

Banavali and colleagues reported on a case of relapsing metastatic angiosarcoma treated with a combination of metronomic oral low-dose chemotherapy (etoposide and cyclophosphamide), celecoxib and PRO (40 mg twice a day) [81]. The patient showed a complete response after two cycles of therapy. After one year of treatment the patient remained on a maintenance treatment of oral cyclophosphamide (50 mg) and PRO (20 mg BID), on alternate days for six additional months after which treatment ceased. The patient relapsed 20 months after initiation of the metronomic treatment and was treated with local palliative radiotherapy and oral thalidomide 100 mg with some response but ultimately died of progressive disease.

Subsequently the same group have reported on a series of seven cases of advanced angiosarcoma treated with a combination of PRO (40 mg BID), weekly vinblastine (i.v. 6 mg/m^2^ to a maximum of 10 mg) and methotrexate (35 mg/m^2^ to a maximum 50 mg) for up to 12 months followed by maintenance of PRO (40 mg BID), oral etoposide (50 mg/day) and oral cyclophosphamide (50 mg/day) for 20 consecutive days in cycles of 30 days [44]. The treatment was well tolerated and showed a 100% response rate, including one complete response and three very good partial responses. Median PFS was 11 months (range 5–24) and OS was 16 months (range 10–30).

Chow *et al* also published a case report in angiosarcoma treated with PRO [82]. The patient presented with a widely disseminated non-metastatic multifocal stage T2 cutaneous angiosarcoma, a diagnosis with a 2-year survival rate of 0%. Treatment with PRO at a dose of 40 mg BID was initiated leading to clinical improvement within a week and a subsequent increase in the dose to 40 mg three times a day. Staining for Ki-67 showed that the PRO monotherapy was associated with a 34% reduction in proliferation rate. Subsequent treatments, administered concurrently with PRO, included 10-weeks of paclitaxel poliglumex (a formulation of paclitaxel designed to increase the therapeutic index of the drug) and radiotherapy. Following cessation of paclitaxel and radiotherapy the patient has continued to take PRO at a dose of 40 mg TID, with clear signs of disease regression and no evidence of metastatic disease.

### Other

Bhattacharyya *et al* reported on the use of the combination of metronomic temozolomide (mTMZ), the COX-2 inhibitor etodolac and PRO in recurrent glioblastoma at ASCO 2014 [83]. A series of 32 patients were randomised to either mTMZ or mTMZ with VT-122 (the combination of PRO 20 mg BID and etodolac 400 mg BID). The median TTP was 5.2 months in the mTMZ arm and 8.8 months in the mTMZ + VT-122 arm. Survival at six months was 40% and 63%, and OS at one year was 12% versus 22% respectively. An update in 2015, also reported at ASCO, with 41 patients included, showed a median OS of 9.2 months versus 17.6 months, and a response rate of 35% versus 57% respectively [84].

The same group also reported on a small (n = 37) single centre open-label trial of gemcitabine and nab-paclitxel (GemNab) with and without VT-122 in patients with locally advanced or metastatic pancreatic cancer [85]. Patients in the GemNab + VT-122 arm (n = 20) were treated with VT-122 for one week prior to commencement of GemNab, and then continuously with GemNab. PFS was 7.2 and 11.8 months, and OS was 10.5 months versus 15.9 months for the GemNab and GemNab + VT-122 arms respectively. Additionally patients in the GemNab + VT-122 arm experienced reduced pain and neuropathy and increased weight gain compared to the GemNab patients.

VT-122 was also used in a small (n = 20), multi-centre, randomised controlled trial in combination with sorafenib in HCC [86]. Median OS was 9.6 months versus 17.2 months in the sorafenib and sorafenib + VT-122 arms respectively.

There has also been some interest in the role of PRO in addressing cancer-related cachexia, a significant cause of morbidity and mortality in late-stage cancer patients [87]. Hyltander *et al* explored influences on resting energy expenditure (REE) in weight-losing cancer patients taking PRO (80 mg BID), the non-selective COX inhibitor indomethacin, morphine or placebo [88]. Patients in the PRO group showed a decrease in REE of around 10% compared to base-line after 5 days of treatment (P < 0.02), whereas there were no significant changes in the other treatment groups or controls. A subsequent study by the same group compared PRO (80 mg/day) and atenolol (50 mg/day) in 10 weight-losing solid tumour patients [89]. While both drugs significantly reduced REE (P < 0.05), PRO treatment, accounting for a decline in heart rate, was significantly more pronounced compared with atenolol (P<0.05).

There has also been some interest in the psychological effects of PRO in cancer patients, for example in reducing the level of emotional distress, measured in terms of the number and rate of intrusive thoughts, associated with a cancer diagnosis [90].

## Clinical Trials

As of 31^st^ May 2016, a number of clinical trials are investigating the anti-cancer uses of PRO. Only trials which are currently open (recruiting or soon to commence recruitment) or on-going are included.

### Feasibility/Phase I

NCT01504126 – A small single arm, open-label (n = 25) trial of PRO, 20 mg BID, with surgery and standard platinum or taxane chemotherapy in ovarian cancer. This is designated as a feasibility study, with a primary end-point of the proportion of patients completing treatment. Treatment commences 48 – 72 hours prior to surgical resection and continues for six cycles of chemotherapy post-surgery.

NCT02013492 – A feasibility study of oral PRO in patients with non-resectable recurrent or metastatic solid tumours. Patients in this open-label study are treated with PRO BID (dose not specified) for four months in the absence of disease progression or unacceptable toxicity. Primary end-points are incidence of toxicity, change in VEGF levels, measurement of impacts on immune response and tumour microenvironment. Secondary outcome measures include one-year PFS and OS.

NCT02897986 (PROVIN) – This planned Phase I dose escalation trial (10, 20 and 30 mg/m² of thrice weekly oral vinorelbine only plus daily PRO 1.5mg/kg/day BID) after completion of the first cycle. Pharmacokinetic analysis of vinorelbine and PRO will be performed. Once the recommended dose of the combination is established four extension cohorts of 9 patients (neuroblastoma, rhabdomyosarcoma, Ewing’s sarcoma, and miscellaneous tumours) will be added. Potential biomarkers (tumour beta-adrenergic receptor expression and beta-tubulin isotypes) will also be evaluated.

NCT02732678 – A dose-finding trial of PRO in combination with metronomic fixed oral cyclophosphamide in patients with locally advanced or metastatic angiosarcoma (PROPAN)

### Randomised – no phase listed

NCT00502684 – A randomised, placebo-controlled trial of peri-operative PRO and etodolac in women undergoing breast cancer surgery. Women in the treatment arm will receive 40 mg of PRO and 800 mg of etodolac starting two days prior to surgery and continuing until three days post-surgery. The trial has a number of biochemical primary outcome measures including immune and angiogenesis-related biomarkers (number and cytotoxic activity of NK cells, levels of NK T-cells, lymphocytes, monocytes and granulocytes; levels of cortisol and VEGF). The primary clinical end-point is the five-year recurrence rate.

### Phase II

NCT01847001 – A Phase II open-label study in newly diagnosed breast cancer patients undergoing neo-adjuvant chemotherapy. The starting dose of PRO is 20 mg BID, but is up-titrated to 40 mg then 80 mg BID. Primary outcomes are PRO compliance during chemotherapy, changes in angiogenesis (as assessed using Diffuse Optical Tomography) and changes in psychological stress levels. Secondary outcomes include adverse event rates and changes in tumour proliferation (Ki-67 staining).

NCT02596867 – A Phase II open-label ‘window of opportunity’ trial in newly diagnosed breast cancer. PRO, at a dose of 1.5 mg/kg BID, is administered for three weeks prior to surgical resection. The primary outcome is a reduction in the proliferative index (Ki-67), secondary outcomes relate to safety, toxicity and adherence.

NCT01988831 – A Phase II randomised, placebo controlled trial in high-risk primary melanoma. Patients at high risk of disease recurrence will receive PRO at a dose assessed by a cardiologist, to a maximum of 160 mg/day. The primary outcome is five-year PFS. [Recruitment currently suspended due to financial reasons].

NCT01857817 – A Phase II randomised, placebo controlled trial of PRO and etodolac in prostate cancer. The drug combination, at a specific dose of 22 mg PRO and 340 mg etodolac, is designated as VT-122, by the sponsor of the trial, Vicus Therapeutics. The dose used in this trial is 22 mg PRO and 340 mg of etodolac twice a day. Primary outcome is change in PSA at 12 weeks. Secondary outcomes include PSA doubling time, PSA progression and time to symptom progression.

NCT01265576 – A Phase II randomised, placebo-controlled trial of VT-122 with sorafenib in hepatocellular carcinoma (HCC) patients at risk of cachexia. The primary outcome is failure-free survival at 6-months. The clinical benefit rate at 6-months is the secondary outcome. Note that a previous trial of VT-122 in NSCLC-related cachexia has yet to report results (trial completed December 2012).

NCT02641314 – A Phase II randomised trial of metronomic chemotherapy and PRO in children and adolescents with recurrent or progressive high risk neuroblastoma (METRO-NB2012). Treatment consists of eight alternating 28-day-cycles of PRO, celecoxib, oral cyclophosphamide, fortnightly i.v. vinblastine, oral etoposide (PCCVE) and of PRO, celecoxib, oral cyclophosphamide, fortnightly i.v. vinblastine (PCCV) followed by five cycles PCCV resulting in a total of 13 cycles (364 days of treatment). The daily dose of PRO is 0.5 mg/kg/day, to a maximum of 120 mg/day, in two divided doses. The primary outcome is to demonstrate the non-inferiority of event free survival (EFS) in comparison to a historical control group. Secondary outcomes include the disease control rate at 6 months and overall survival at 12 months.

ACTRN12615000889550 – A Phase II randomised study of perioperative PRO vs placebo on gene expression in newly diagnosed breast cancer. The treatment group will receive 7 days of pre-operative PRO (40 mg BID days 1 – 3, 80 mg BID days 4 - 8) prior to and including the day of surgery and then will be titrated off PRO over two days in the post-operative period. The primary outcome is tumour gene expression for each of 20,000 genes at baseline and at surgical resection.

ACTRN12612000852853 – A Phase II randomised controlled trial of peri-operative PRO and etodolac in colorectal cancer patients undergoing surgical excision of the primary tumour. The primary outcome is a reduction of two-year rate of recurrence and distant metastases.

### Phase III

NCT00888797 – This is a sister trial to NCT00502684 and is a Phase III randomised, placebo-controlled trial of peri-operative PRO and etodolac in colorectal cancer patients undergoing resection. Patients in the treatment arm receive etodolac 800 mg BID for the entire intervention period, PRO 20 mg BID for 5 pre-operative days, 80 mg BID on the day of surgery, 40 mg BID for the first postoperative week, 20 mg PO BID for the second postoperative week. The primary clinical end-point is the rate of local and distant recurrence rate at three years. Secondary end points are immune-related.

EudraCT 2014-003671-30 – This open label Phase III trial in patients with VHL syndrome will assess the efficacy of PRO, at a dose of 2 mg/kg, in controlling the growth of papillary and juxta-papillary retinal hemangioblastomas. The primary endpoint is a reduction in the number and size of retinal hemangioblastomas at 12-months.

## Mechanisms of Action

PRO is a non-selective beta-adrenergic receptor antagonist, with a similar binding affinity for beta1- and beta2- and a much lower affinity (approximately 100-fold) for the beta3-adrenoreceptor [91]. In this respect PRO has a similar selectivity to some other clinically used beta-blocker drugs, particularly pindolol and carvedilol and there exists some evidence that these particular drugs may also have potential in repurposing [92–93]. There are a number of distinct putative mechanisms of action that have been investigated in relation to the anticancer effects of PRO, many of them associated with the beta2-adrenoreceptor pathway and which may be particularly important in the context of the metastatic process [94].

### Proliferation

Investigations of the influence of beta-adrenergic signalling on cellular proliferation extend back more than fifty years. In 1961 Selye, Veilleux and Cantin reported that rats chronically treated with the beta-adrenergic agonist isoproterenol displayed excessive growth of salivary glands, most likely due to an increased mitotic rate [95]. Barka subsequently confirmed that isoproterenol increased the rate of mitosis and triggered DNA synthesis [96]. The converse, a reduction of proliferation due to PRO was also reported in the same era, for example Iwata, Kariya and Fujimoto showed that in a ciliated protozoan beta-adrenergic blockade, with PRO and other agents, was associated with a reduced rate of growth [97]. It has since been shown that PRO is able to inhibit the increase in cancer cell proliferation associated with catecholamines or isoproterenol in a number of cancer cell types [56, 98–100].

In a comparison between PRO and the selective beta adrenergic receptor antagonists atenolol (beta1) and ICI118-551 (beta 2), Işeri and colleagues showed that PRO and ICI118,551 were more potent in reducing the proliferation, migration, and invasion of non-stimulated breast (MCF7), colon (HT-29), and hepatocellular (HepG2) cancer cells than atenolol [101].

In contrast, there have also been contradictory results, for example for breast cancer where Pérez Piñero *et al* have reported that beta adrenergic agonists isoprenaline and salbutamol reduced breast tumour growth in animal models, and that PRO treatment reversed this inhibition [102] and other authors have reported a decrease in proliferation in breast cancer cell line, for example in the MCF7 cell line [101].

Bernabé *et al* showed that OSCC proliferation in response to increased beta2 adrenergic signalling was mediated by IL-6 [61]. In addition to showing increased IL-6 production from OSCC cells lines in response to norepinephrine or isoproterenol, neutralising IL-6 using antibodies inhibited the increase in proliferation rate. PRO was also shown to inhibit the increased proliferation rate induced by IL-6. A decrease in circulating IL-6 levels in response to PRO has been reported in a murine melanoma model, concomitant with a lower level of metastatic growth compared to untreated controls [103].

### Migration and Invasion

Strell *et al* have shown that PRO is able to abrogate the norepinephrine-induced increase in migratory activity of a range of breast cancer cell lines [28–29].

Degradation of the extra-cellular matrix is a key factor in tumour progression and the metastatic cascade, with the matrix metalloproteinases (MMPs) playing a central role in the tissue remodelling process [104–105]. Sood *et al* reported that MMP-2 and MMP-9 were highly expressed during the norepinephrine-induced increase in ovarian cancer invasiveness [39]. Furthermore they showed that PRO treatment could reduce the rate of tumour growth and infiltration in vivo. A similar inhibition of the upregulation of MMP-2 and MMP-9 by PRO has also been reported in nasopharyngeal carcinoma [59], pancreatic cancer [50, 53], gastric adenocarcinoma [66], melanoma [106], prostate cancer [107] and of MMP-9 in medulloblastoma [108] and infantile hemangioma [109–110].

Pon *et al* described a beta2 adrenergic receptor activated feed-forward loop driving the invasiveness of the highly metastatic MDA-MB-231-HM breast cancer cell line [111]. A range of beta2 adrenergic receptor agonists caused a dose dependent increase in cAMP and Ca^2+^ signalling and a decrease in phosphorylated ERK, which was competitively inhibited by PRO and the selective beta2 adrenergic receptor antagonist ICI-118551. The increased invasion of the MDA-MB-231-HM cell line was shown to be associated with the positive feedback between cAMP and Ca^2+^ but independent of the effect on pERK.

Tissue remodelling is one of the key steps in the process of epithelial-mesenchymal transition (EMT) which is important in metastasis [112]. Shan *et al* showed that the human gastric cancer cell lines BGC-823 and SGC-7901 underwent morphological changes typical of EMT, and showed increased invasiveness, in response to co-culture with norepinephrine via a beta2-adrenergic signalling pathway [113]. Zhang *et al* showed a similar association between increased catecholamine signalling and initiation of EMT in HT-29 and A549 colorectal cancer cell lines [114]. Additionally they showed that TGF-β1 mediated the norepinephrine induced EMT process and that PRO, at a concentration of 10 μM, inhibited the increase in TGF-β1 and partially decreased the migration and invasiveness of norepinephrine-treated cells.

Related to the degradation of the extra-cellular matrix and the EMT process is the formation of tumour cell invadopodia, which are actin structures formed on the surface of cancer cells believed to be active in the secretion of MMPs [115]. Creed *et al* have shown that beta2 adrenergic signalling increased the rate of invadopodia formation in human and murine breast cancer cell lines in vitro, and that PRO inhibited this increase [116].

Also important in the metastatic process is cell-cell adhesion, and here too there is evidence that adrenergic signalling is active. Rap1B is a small GTPase that increases cell-cell adhesion. There is evidence to suggest that loss of Rap1B at the plasma membrane decreases cell-cell adhesion and may promote a metastatic phenotype [117–118]. MDA-MB-231 breast cancer cells showed reduced cell-cell adhesion in response to isoproterenol and an increase in cell migration, whereas treatment with PRO reduced the level of migration [119].

The process of metastasis depends also on the creation of a ‘metastatic niche’, in addition to the properties of the primary cancer cells [120–121]. Campbell at al showed that in a mouse model restraint-induced stress or exogenous isoproterenol promoted MDA-231 breast cancer cell colonization of bone via adrenergic signalling effects on the bone marrow stromal compartment [31]. This effect was via upregulation of RANKL, which increased the number of osteolytic lesions in response to catecholamine signalling, and that this effect could be reversed by PRO or the RANKL inhibitor denosumab.

The role of the lymphatic system in metastasis has become increasingly recognised in recent years [122]. Le *et al* showed that chronic stress induced a remodelling of the tumour lymphatic vasculature, including an increase in lymphatic vessel density and vessel dilation leading to elevated levels of lymphatic draining [123]. These effects were sensitive to beta-adrenergic signalling and could be enhanced by isoproterenol or inhibited by PRO treatment of BALB/c mice injected with MDA-MB-231 breast cancer cells. The stress-induced remodelling was mediated by VEGFC production, which was induced by COX-2/PGE_2_. PRO has also been explored as a treatment option for congenital lymphangioma [124].

### Apoptosis

Zhang *et al* showed that PRO limited the expansion of the PC-2 pancreatic cancer cell line and that this was due to an increased rate of apoptosis [51]. The pro-apoptotic action of PRO was found to be via blockade of the beta2-adrenergic receptor rather than beta1, as shown by the increased level of apoptosis induced by the selective beta2 antagonist butaxamine and the reduced level due to the beta1 blocker metoprolol. Chin and colleagues also showed that in colorectal cancer cell lines PRO was associated with cell cycle arrest and apoptosis [57].

Liao *et al* reported that in vitro PRO concentrations of 200 μM induced cell cycle arrest and apoptosis in gastric carcinoma cell lines. Apoptosis was associated with a decrease in levels of NF-κB, VEGF, COX-2, MMP-2 and MMP-9 expression [66].

The pro-apoptotic activity of PRO was also investigated by Wolter and colleagues in their work in head and neck squamous cell carcinoma (HNSCC) cell lines with differing p53 status [62]. PRO treatment was shown to cause apoptosis irrespective of p53 status and was related to down-stream activity of p63 and p73, both p53-family proteins. Following PRO treatment there was evidence of downregulation of the anti-apoptotic ΔNp63a and induction of the pro-apoptotic TAp73b in both SCC9 and SCC17a cell lines. Some of the same authors also investigated the pro-apoptotic effect of PRO on neuroblastoma cell lines and showed that treatment increased expression of p53 and p73 [47].

### Angiogenesis

The relationship between adrenergic signalling and angiogenesis was first elucidated in the late 1990s, when it was shown that beta adrenergic signalling by norepinephrine induced increased levels of VEGF expression in brown adipose tissue [125–126].

Lutgendorf and colleagues showed that beta adrenergic agonists increased the expression of VEGF in two ovarian cancer cell lines (EC and SKOV3), and that PRO, at a concentration of 1 μM, blocked this increase [38]. This finding suggested a putative link between behavioural stress and enhanced tumour growth via increased angiogenesis. In vivo work using a murine model of ovarian cancer showed that chronic behavioural stress was associated with increased tumour growth and vascularisation and enhanced expression of VEGF, MMP-2 and MMP-9. In particular beta-adrenergic activation of the cAMP-PKA signalling pathway was identified as a major mechanism by which behavioural stress enhanced tumour angiogenesis [127]. In subsequent work some of the same authors showed that surgical stress induced increased beta-adrenergic signalling in a murine ovarian cancer model and that this was associated with increased rates of tumour growth and tumour angiogenesis [40]. Perioperative PRO was shown to inhibit the surgical stress-induced increase in VEGF expression and the consequent increase in angiogenesis and tumour growth.

An anti-angiogenic effect of PRO, via down-regulation of VEGF has also been shown in a range of cancer cell lines including nasopharyngeal carcinoma [59], melanoma [128], pancreatic cancer [50], leukaemia [23], head and neck squamous cell carcinoma [62] and infantile hemangiomas [129–130]. Pasquier *et al* showed that the combination of beta-blockers, including PRO, with vincristine was associated with increased survival and reduced angiogenesis in a mouse model of neuroblastoma [45].

Other mechanisms may also play a role in the anti-angiogenic effects of PRO. For example, Annabi *et al*, following initial reports that PRO was effective in infantile hemangiomas [131], investigated PRO activity in human glioblastoma biopsy samples [60]. It was reported that PRO down-regulated endothelial MMP-9 expression and reduced the rate of human brain microvascular endothelial cells tubologenesis, potentially reducing tumour angiogenesis.

Park *et al* showed that hypoxia-inducible factor 1α (HIF-1α) expression is also upregulated by norepinephrine, via the cAMP/PKA/Akt/p70S6K pathway, in addition to VEGF, and that it plays a key role in the angiogenic process [132]. Silencing of HIF-1α reduced the norepinephrine-induced increase in VEGF expression and capillary tube formation. Furthermore PRO pre-treatment abrogated the effect of adrenergic signalling on HIF-1α, VEGF and angiogenesis. Similar results, using a beta2-adrenergic receptor antagonist (ICI118 551), have been shown in vitro and in vivo in a murine pancreatic cancer model [133].

In addition to VEGF, MMP-2 and MMP-9, Liao *et al* showed that isoproterenol increased levels of COX-2, in gastric cancer cell lines, while PRO significantly reduced expression (P < 0.05) [66]. The COX-2/prostaglandin E2 (PGE_2_) pathway is also known to be involved in cancer-associated angiogenesis [134–135]. These effects were partly due to activation of the activation of NF-κB pathway [66]. Ciccarelli and colleagues showed that genetic deletion of b2-adrenergic receptors impaired angiogenesis in a mouse model, and that isoproterenol induced IκBα degradation and enhanced NF-kB transcriptional activity in a time-dependent manner [136].

### Treatment Sensitisation

Early in vitro work showed that PRO had the potential to revert the drug resistant phenotype in different cell lines, including doxorubicin-resistant P388 murine leukaemia [18] and human multi-drug resistant (MDR) CEM leukaemia [137] for example. But results were cell line and drug specific, for example PRO seemed to have little impact on the cisplatin sensitivity of NSCLC cell lines [138].

In addition to chemosensitisation there has also been some preclinical work investigating the relationship between PRO and radiation. Liao *et al* showed that pre-treatment of the human gastric adenocarcinoma (HGC) cell lines BGC-823 and SGC-7901 with PRO, at a concentration of 50 μM for 24 hours, increased the effect of radiotherapy on cell viability in vitro [67]. Similarly, Wolter *et al* assessed the impact of PRO on HNSCC cell lines and showed that it enhanced the effect of radiation, in addition to displaying evidence of synergy with cisplatin [62]. We may also note the concurrent use of PRO and chemoradiotherapy in one of the case reports of PRO use in angiosarcoma [82].

The mechanism for increased radiosensitivity may be related to the NF-κB/COX-2/PGE_2_ pathway inhibition that a number of investigators have reported [36, 52, 67]. Evidence exists to suggest that elevated COX-2 expression may confer increased radiation resistance in some cancer cell lines [139–141].

Pasquier *et al* investigated the synergism of PRO with paclitaxel and 5-FU, both in vitro and in vivo [30]. In vitro analysis of a number of human cancer and non-cancer lines showed a range of synergistic, additive, sub-additive and antagonistic effects on cell proliferation depending on dose, cell line and chemotherapy drug. The synergistic effects were shown to be due to an enhancement of the anti-angiogenic effects of the chemotherapy drugs by low concentrations (10 μM) of PRO. In vivo a murine orthotopic triple negative breast cancer (MDA-MB-231) xenograft model was used with each chemotherapy drug. Four treatment groups of tumour-bearing mice were used, control (saline-treatment), paclitaxel alone (20 mg/kg, 3 days a week for 3 weeks), PRO alone (10 mg/kg, 5 days a week for 5 weeks) or the combination of paclitaxel and PRO. The same protocol was used with 5-FU, and the dose of the drug was 5-FU alone (30 mg/kg, 3 days a week for 5 weeks). The combination treatments produced significantly improved median survival times both for paclitaxel (125 days vs 70 for paclitaxel alone, or 47 for control, P = 0.0005) and 5-FU (56 days vs 47 for 5-FU or 44 for control, P = 0.0005). Subsequently the same group published results which showed PRO synergised with vincristine in a murine model of neuroblastoma [45].

There are still other examples of PRO acting to improve cancer cell sensitivity to drug treatment enhancing the effect of rapamycin on human prostate cancer PC3 cells [49], reverting resistance to trastuzumab in HER2 breast cancer [32], inhibiting the stress-related reduction of sunitinib activity in colorectal cancer in an in vivo model [142] and sensitising thyroid cancer cells to the targeted BRAF-V600E inhibitor vemurafenib [72].

### Immunological

A number of immune-related mechanisms of action have also been outlined as important in the anti-cancer effects of PRO. These effects are primarily mediated by the effects of sympathetic nervous system (SNS) signalling on different populations of immune cells, the tumour microenvironment and, in some cases, directly on cancer cells. An important aspect of this complex relationship is the link between psychological stress and immune response, a key concern within the field of psychoneuroimmunology and increasingly important in the context of cancer [143]. While there are a number of papers which review the effect of physical and psychological stress on the immune system, for example [144–145], the primary focus in this paper is on direct evidence of the role of PRO, and where relevant, other beta adrenergic receptor antagonists.

Teshima *et al* showed that in C3H/H and AKR mice PRO, at a dose of 5 mg/kg, was able to increase the phagocytic activity of macrophages (P < 0.05) and inhibit the reduction of phagocytic activity induced by physical restraint-induced stress (P <0.01) [146].

Shakhar and Ben-Eliyahu showed that the beta-adrenergic agonist metaproterenol induced a dose-dependent transient increase in natural killer (NK) cell numbers within 10 minutes of administration in F344 rats (P < 0.0001) [24]. It should be noted that although it did not reach significance, the time course of NK numbers showed that the initial increase subsided within one hour and before falling below base-line values by three hours and returning to base-line at five hours. Blood NK activity was depressed by metaproterenol (P < 0.03), but this was inhibited by prior administration of PRO (at doses in the range 0.1 – 0.5 mg/kg) or nadolol. Additional experiments showed that nadolol, like PRO a non-selective beta-adrenergic receptor antagonist, reduced the number of lung-retained NK-sensitive MADB106 breast cancer cells and the number of lung metastases.

Subsequently Benish *et al* showed that surgical intervention (laparotomy) prior to inoculation with MADB106 cells in F344 rats was significantly (P < 0.05) associated with increased the rate of lung tumour cell retention (LTR) [25]. This increase in LTR was attenuated by pre-surgical treatment with COX-2 inhibitors (indomethacin, etodolac, and celecoxib). Pre-surgical PRO, at a dose of 1.5 mg/kg and 4.5 mg/kg, also significantly reduced the LTR rate compared to untreated controls. The combined treatment of etodolac (12.5 mg/kg) and PRO (1.5 mg/kg) was more effective than either single treatment and completely inhibited the effect of surgery. Notably the combined treatment reversed the surgically induced reduction in NK cell numbers and per-cell cytotoxicity. These deleterious impacts on NK cells were later shown to be associated with reduced FasL and CD11a expression post-surgery, and that the combined etodolac PRO treatment counter-acted these effects [36].

Kalinichenko *et al* investigated the effect of norepinephrine on cytotoxic T lymphocytes (CTL) using a MOPC-315 plasmocytoma model in BALB/c mice [147]. Results showed that norepinephrine inhibited CTL generation via a reduction of TNF-α expression in CD4+ and CD8+ T cells and F4/80+ activated macrophages, a result confirmed by the addition of exogenous TNF-α. Ex vivo use of PRO, at a concentration of 1 μM, completely reversed the effects of norepinephrine on TNF-α and CTL generation. Sloan *et al* also identified stress-sensitive CD11b+F4/80+ macrophages as being implicated in the metastatic process in the same mouse model [27].

Wu *et al* showed that stress induced by social isolation in Balb/c mice injected with colon 26-L5 carcinoma cells increased the rate of liver metastases compared to unstressed controls (P < 0.05) [148]. This was shown to be associated with a reduction in thymus weight, reduction in thymocytes and reduction in cytolytic activity of NK cells. Subsequent research by the same laboratory replicated these findings in additional mouse models and also showed that over-crowding induced similar stress-related thymic atrophy [33]. Additionally it was shown that PRO, at a dose of 30 ppm, slowed the rate of tumour growth of the over-crowded group to below that of the unstressed controls.

Kanemi *et al* showed that stressed induced by physical restraint in C57BL/6 mice resulted in a significant increase in epinephrine levels (P < 0.001) which returned to base-line values within one hour [149]. Lymphocyte numbers in blood and lungs were depressed (P <0.001) by restraint, but numbers returned to base-line within four hours of cessation of restraint. Lung NK cell numbers were also reduced by restraint-induced stress (P < 0.01), as were all other lymphocyte subsets assessed (CD8+, CD4+, B cell and NKT cells). PRO, at a dose of 20 mg/kg, administered prior to restraint was shown to reverse the reduction in NK cell numbers compared to untreated controls but had no effect on other lymphocyte sub-sets in the lungs.

In contrast, Tarr *et al* showed that repeated social disruption stress (induced by repeatedly introducing aggressive mice into cages of non-aggressive mice), both increased splenic NK cell numbers, NK cytotoxic activity and the expression of activation markers, both immediately and 14 hours after stress [150]. Administration of PRO (10 mg/kg) reduced the ‘priming’ of these NK cells at 14 hours. The authors propose an evolutionary explanation for these findings, suggesting that the priming occurs in order to prepare the host for pathogenic insult during stressful ‘fight or flight’ episodes. However, it has been argued that the complexity of multiple immune compartments, NK cell lineages and complexity of following cell populations in vivo over extended time periods makes interpretation of these results difficult [151].

Catecholamines are also known to impact the immune response via down-regulation of interferon gamma (IFN-γ) production [152]. Khalili *et al* showed that PRO and a HSP-70-rich tumour lysate vaccine synergised to increase IFN-γ production in a murine model of fibrosarcoma [153]. Treated animals showed lower rates of tumour growth (P < 0.01) and increased level of CTL activity (P < 0.05).

Lymphocytes are also known to secrete catecholamines, with potential downstream impacts on immunity. Huang *et al* investigated the effect of lymphocyte-derived catecholamines on the differentiation and function of T helper (Th) cells, suggesting that they shifted the Th1/Th2 balance in the direction of greater Th2 polarisation [154–155]. Panina-Bordignon *et al* had earlier suggested that beta2-adrenergic signalling inhibits production of IL-12, thereby promoting Th2 differentiation and inhibiting the Th1 development associated with anti-tumour immunity [156].

Myeloid derived suppressor cells (MDSC) are implicated in the dysfunctional immune response to cancer and are considered a negative prognostic marker in some cancers [157–158]. Jin *et al* used BALB/c mice to show that chronic stress induced an immunosuppressive state associated with an accumulation of CD11b+Gr1+ MDSCs in the bone marrow [159]. In line with previous reports from other groups [160], the data showed that the COX-2/PGE_2_ axis a central role in this accumulation. In addition there was evidence that stress-related catecholamines were also implicated, and that PRO (10mg/kg) partially reversed the accumulation of MDSCs, both in terms of cell numbers (P < 0.001) and proportion (P = 0.018).

T-regulatory (T-reg) cells are another population of immune cells associated with tumour-associated immune dysfunction. Zhou *et al* studied the impact of PRO and the COX-2 inhibitor parecoxib on T-reg numbers in breast cancer patients undergoing radical mastectomy [161]. Patients were assigned to control, PRO, parecoxib and PRO + parecoxib groups. Patients in the PRO group received 20 mg TID starting from day of surgery until third post-operative day. The parecoxib group received 40 mg per day, IV, from day of surgery to second post-operative day. Patients in the combination group received both treatments at the same dose and schedule. Results showed that surgery was associated with an increase in epinephrine, norepinephrine, PGE_2_ levels and T-reg numbers. Treatment with PRO or PRO + parecoxib attenuated the increase in T-reg numbers though there was no additive or synergistic effect of the parecoxib compared to PRO alone. Ex vivo analysis showed that PRO also reduced the immunosuppressive effect of T-reg cells compared to controls.

### Other

In addition to the main mechanisms that have been outlined about, there are also a number of other possible mechanisms of action which have been described in the literature, some of which may be unrelated to the beta-adrenergic receptor antagonist activity of PRO.

Epidermal growth factor receptor (EGFR) signalling plays a central role in many cancer types and is a major drug target [162]. Disrupted endocytic trafficking is implicated in the process by which tumours gain self-sufficiency in growth signals by delays in the inactivation of multiple growth factor receptors, including EGFR [163–164]. It has been proposed by Shaughnessy *et al* that a strategy for inhibiting EGFR function may be to interfere with the endocytic process directly rather than directly targeting receptor-ligand binding or tyrosine kinase activity [165]. Inhibition of phosphatidic acid phosphohydrolase (PAP) has been shown to cause a reversible trafficking of inactive (empty) EGFR from the cell surface to endosomes, thereby restricting receptor availability to ligands [166]. PRO is a known inhibitor of PAP [167], and has been shown to reduce the cell viability of EGFR-dependent cancer cell lines [165].

Kang *et al* investigated the impact of PRO on glucose uptake in breast cancer cell lines (4T1, MDA-MB-231 and MCF-7) [168]. In vitro analysis showed that while glucose transporter-1 (GLUT-1) was relatively unaffected in all cell lines by PRO, hexokinase-2 (HK-2) was sensitive to PRO at a concentration of 50 μM. In vivo results using the 4T1 mouse breast cancer line showed that tumours were also sensitive to PRO at a dose of 10 mg/kg, and that this was associated with a reduction in HK-2 expression. PRO may therefore have a metabolic impact on tumour growth.

PGE_2_ is a key inflammatory molecule with multiple effects in cancer, including effects on immunity, angiogenesis, proliferation and apoptosis [169]. As has already been shown, these diverse effects are also implicated in some of the mechanisms of action of PRO. Nagaraja *et al* sought to investigate the relationship between beta adrenergic signalling and PGE_2_ in more detail using primary ovarian cancer cells from patients [170]. Tumour samples from depressed patients showed significantly higher levels of PGE_2_ and PGF2α, tumour samples from mice bearing Skov3-ip1 ovarian tumours also showed elevated levels of PGE_2_. Analysis showed that norepinephrine increased the secretion of PGE_2_ from tumour cells. Furthermore PRO was also shown to inhibit the catecholamine-induced increase in PGE_2_ in Skov3 and HeyA8 ovarian cancer cells.

Platelets play a complex role in tumour progression and metastasis via the release of pro-angiogenic factors, a role in subverting anti-tumour immunity by ‘cloaking’ tumour cells from NK cells and a role in establishing the metastatic niche [171]. The anticancer effects of aspirin may be related to anti-platelet effects via irreversible inhibition of COX-1 [172–173], and there is also some evidence that other anti-platelet agents, such as low molecular weight heparins and dipyridamole may also have anti-cancer or anti-metastatic activity [174–176]. Beta-adrenergic receptor antagonists are also known to have effects on platelet aggregation and a meta-analysis published in 2014 showed that they decreased platelet aggregation by 13% (95% CI = 8 - 17%, standardised mean difference=-0.54, 95% CI = -0.85 – -0.24, P < 0.0001) [177]. In particular non-selective lipophilic beta-blockers (including PRO) decreased platelet aggregation more than selective non-lipophilic beta-blockers. A small randomised cross-over trial in moderate essential hypertension compared PRO (40mg TID) to atenolol (100 mg/day) confirmed that the number of circulating platelet aggregates decreased significantly with PRO (0.99±0.19) in comparison with both atenolol (1.41±0.70; P = 0.004) and baseline (1.59±0.94; P = 0.002) [178].

Finally, beta-adrenergic signalling is at the intersection between psychological states and physiology. As has been previously mentioned stress arising from social interactions has been shown to have negative effects on proliferation, invasion, metastasis and anticancer immunity. However, it should be noted that not all forms of social stress are necessarily negative – clinical psychology differentiates between positive stress (eustress) and negative stress (distress) with differing physiological correlates [179]. Cao *et al* showed that a murine model of eustress, related to living in an enriched environment (increased levels of inanimate stimulation, social stimulation and physical exercise), was associated with reduced rate of B16 melanoma growth compared living in a control environment [180]. This reduction in tumour growth rate was associated with down-regulation of hypothalamic brain-derived neurotrophic factor (BDNF) and increased production of leptin in adipocytes via beta-adrenergic signalling. PRO, at a dose of 0.5 g/l in drinking water, inhibited the protective effect of the enriched environment.

## Our Take

The evidence outlined above, and summarised in Table 1, suggests that PRO has a number of distinct anti-cancer effects which may be of therapeutic value in different clinical settings. While there is evidence that other drugs in the same class as have anti-cancer effects, for example carvedilol and nebivolol [45], PRO has both the widest range of evidence and extensive clinical use, therefore pragmatic reasons suggest that it be prioritised for further clinical research. Needless to say, the repurposing of other beta-blockers may also carry great potential in the same or other settings/tumours. For PRO the evidence in angiosarcoma, anti-metastatic effects in breast and ovarian cancers and the effect on the rate of distant metastases following surgical intervention are particularly noteworthy. In all future trials, it will be important to assess plasma concentrations of propranolol especially when a direct effect on tumour growth is desirable.

### Angiosarcoma

Angiosarcoma is a rare and aggressive soft tissue sarcoma arising in vascular endothelial cells, and is a disease with poor prognosis and reported five-year overall survival rates in the range 30% - 40% [181]. Although no standard treatment exists, the majority of patients are treated with surgical resection, chemotherapy (doxorubicin or paclitaxel most commonly) and radiotherapy [182]. Given the high unmet needs in this disease, the case reports showing some benefit to patients using PRO in combination with other agents are noteworthy [44, 81, 82], along with responses to propranolol observed in other vascular tumours [41, 183–184]. Clinical trials are urgently required to confirm the efficacy of these combinations with PRO.

### Anti-metastatic Agent

Metastatic disease remains the primary cause of cancer-related mortality and therefore the search for anti-metastatic agents is of considerable value [185]. As outlined previously, PRO has multiple mechanisms of action which may have some impact on the metastatic process, including reduction in the rate of invasion, down-regulation of angiogenesis and lymphangiogenesis, inhibition of tissue remodelling and down-regulation of COX-2/PGE_2_ expression. Data from a number of in vivo models has shown that PRO may reduce the rate of metastasis in breast and other cancers via direct effects on beta-adrenergic signalling [27, 36, 48]. The addition of PRO to standard of care for non-metastatic cancers may therefore be a strategy to reduce the rate of metastatic spread.

### Perioperative Intervention

Data from both retrospective analyses of patient outcomes and from animal models suggest that surgical intervention may be associated with distant metastases [186–187]. The ‘wound healing response’ due to the surgical incision initiates a cascade of inflammatory events that lead to suppression of cell-mediated immunity and an increase in pro-angiogenic signalling [188–191]. However, there is now a growing level of interest in targeting some aspects of this post-surgical response so as to reduce the risk of metastatic spread [192–193]. Options for such peri-operative interventions include choice of anaesthesia [194–195], the use of drugs which target the COX-2/PGE_2_ pathway such as ketorolac [196–198] and diclofenac [199–200], and the H2RA cimetidine [201–202]. As should be clear from the results previously outlined, beta-adrenergic signalling is also implicated in the post-surgical metastatic process and numerous in vivo studies have reported that peri-operative PRO is associated with a reduced rate of metastases [40]. Of note the combination of PRO with a COX-2/PGE_2_ inhibitor, such as ketorolac or etodolac, has the potential to show synergism in a peri-operative setting and warrants additional investigation. This is particularly the case in those cancers in which post-surgical distant metastases are a frequent occurrence, including breast cancer, osteosarcoma, head and neck cancers, upper GI cancers, NSCLC and ovarian cancer.

### Other Cancers

While we have outlined areas where there is a particularly compelling case to be made for further clinical study, there is also sufficient evidence to suggest that there may be some value in assessing the potential benefit of PRO in other cancers. The ubiquity of catecholamine signalling and the apparent expression of adrenergic receptors in multiple tumour types would suggest that the effects of PRO may extend, ultimately, to a much larger number of cancer types than has hitherto been suggested [203–204]. In the meantime there is a good level of evidence for further investigation in pancreatic cancer and neuroblastoma, particularly in combination with other agents, including repurposed drugs such as ketorolac or etodolac.

### Psychological Stress

We note that the data for a pro-apoptotic effect of PRO on primary tumour growth comes primarily from in vitro studies which use high concentrations which may not reflect physiologically achievable levels. In many respects this is similar to the case with some NSAIDs, such as diclofenac, where the evidence is that the apoptotic effects are not physiologically achievable, and that therefore the anti-cancer effects are related to aspects of the host environment rather than directly on primary cancer growth [205]. Beta adrenergic signalling is central to the intersection between psychosocial stress and cancer, as evidenced in numerous animal models and epidemiological data. A variety of stress reduction techniques have been clinically investigated, including mindfulness meditation [206], cognitive behavioural stress management [207] and even communal singing [208]. Therefore, while the data for an effect on primary tumour growth may be limited in contrast to the results supporting an anti-metastatic effect, there is reason to believe that beta blockade may be beneficial in terms of psychological effects in addition to physical effects on the host environment.

### Next Steps

The current level of clinical trial activity, which is relatively high for a repurposed drug, testifies to the strong level of clinical evidence and it is to be hoped that positive reports from these trials will be forthcoming in the future.

The data are strongest for clinical trials of PRO, in combination with other agents, in the following cancer types:

- Angiosarcoma
- Breast cancer
- Ovarian cancer
- Pancreatic cancer
- Neuroblastoma

The peri-operative use of PRO in combination with ketorolac or etodolac is also of interest in the following cancers:

- Osteosarcoma
- Head and neck cancers
- Oesophageal cancer
- Breast cancer
- Ovarian cancer
- Non-small Cell Lung Cancer
- Pancreatic cancer

## Conclusion

There is a significant volume of data from in vitro, animal and human studies to indicate that there are multiple clinically relevant anti-cancer effects associated with PRO. This data has been summarised and presented to make the case that PRO is a very strong candidate for repurposing as an anticancer agent. In particular the potential for synergistic interactions with other drugs has been outlined, including repurposed COX-2/PGE_2_ inhibitors and a range of chemotherapeutics at both metronomic and standard dosing. The anti-metastatic properties of PRO may be particularly valuable to exploit during surgical intervention, and a number of possible combinations with other agents is discussed in this setting.

## Author Contributions

Primary author: Pan Pantziarka. Contributing authors: Ilse Rooman, Vidula Sukhatme, Gauthier Bouche, Lydie Meheus, Vikas P. Sukhatme. All authors read and approved the final manuscript.

## Competing Interests

The authors declare that they have no competing interests. All the authors are associated with not for profit organisations that aim to repurpose drugs for oncology treatments. VPS is also a scientific advisory board member of Berg Health and Mitra Biotech and a consultant to Roivant Sciences.

## Figures and Tables

**Table 1.** Summary of evidence by cancer type.

Cancer Type	In Vitro	In Vivo	Case Report/Trial
**Angiosarcoma**	[43]	[43]	[44, 81, 82] NCT02732678
**Breast**		[24–31]	[79] NCT01847001, NCT02596867, NCT00502684 ACTRN12615000889550
**Colorectal**	[55, 57]	[58]	NCT00888797 ACTRN12612000852853
**Gastric**	[66, 67]		
**Glioblastoma**			[83, 84]
**HCC**			[86] NCT01265576
**Leukaemia**	[21, 60]	[22]	
**Melanoma**	[34, 35]	[33, 36]	NCT01988831
**Multiple myeloma**	[70]		
**Nasopharygeal**	[59]		
**Neuroblastoma**	[45, 47]	[46, 47]	NCT02641314 NCT02897986
**NSCLC**	[68]		
**Oral SCC**	[61, 62]		
**Ovarian**	[38]	[39, 40]	NCT01504126
**Pancreatic**	[50–52]	[53, 54]	[85]
**Prostate**	[48]		NCT01857817
